# Operationalizing Healthspan as an Outcome for Clinical Trials in Geroscience

**DOI:** 10.1093/geroni/igab046.1340

**Published:** 2021-12-17

**Authors:** Jamie Justice

**Affiliations:** Wake Forest School of Medicine, Wake Forest School of Medicine, North Carolina, United States

## Abstract

Efforts targeting biological aging pathways are advancing interventions which could extend healthy lifespan. Design of clinical trials to test such interventions necessitates an operational definition of healthspan, such as slowed accumulation or progression of multiple chronic diseases, functional decline, and disability. In this talk we explore these composite measures of healthspan proposed as outcomes for clinical trials in aging. This will be examined in example cases including multimorbidity and deficit accumulation frailty indices in an 8‐Year intensive lifestyle intervention trial, and an update on multimordbity, functional, and biomarker endpoints in the trial Targeting Aging with MEtformin (TAME). Through these examples we will explore issues related to effect sizes and statistical challenges related to composite endpoints. Finally, we will discuss the role existing and emerging biomarkers of aging in clinical trials in geroscience and summarize evidence linking biomarkers to clinically meaningful outcomes.

